# 

**Published:** 1879-03

**Authors:** 


### CONTENTS.

ORIGINAL.

ART. I.—The importance of Chemical Analysis in cases of Obscure Diagnosis. Read before the Buffalo Medical Association. By C. A. Doremus, M. D., Ph. D., Prof. of Chemistry in the Buffalo Medical College, 281

ART. II.—A case of Fistula in Ano, with a few practical remarks regarding the Surgical Operations for its relief; as also the action of the Sphincter Tertius described by Hyrtl. By T. G. Comstock M. D., St. Louis. Mo., ... 294

ART. III.—Clergyman's Sore Throat.—Chronic Follicular Pharangitis. By H. S. Davis, M. D., ... 302

ORIGINAL.

ART. IV.—Suits against Surgeons. By George M. Blake, M. D., Dansville, N. Y., ... 309

EDITORIAL.

The Commencement in the University of Buffalo, ... 316

Meeting of the Alumni Association of Buffalo Medical College, ... 318

Original Department of this Journal, ... 318

Call for Convention of Medical Colleges, ... 319

Books Reviewed, ... 319

Books Received, ... 320

